# Variant effect predictors: a systematic review and practical guide

**DOI:** 10.1007/s00439-024-02670-5

**Published:** 2024-04-04

**Authors:** Cristian Riccio, Max L. Jansen, Linlin Guo, Andreas Ziegler

**Affiliations:** 1 Cardio-CARE, Medizincampus Davos, Herman-Burchard-Str. 1, Davos Wolfgang, 7265 Davos, Switzerland; 2 Swiss Institute of Bioinformatics, Lausanne, Switzerland; 3 Center for Population Health Innovation (POINT), University Heart and Vascular Center Hamburg, University Medical Center Hamburg-Eppendorf, Hamburg, Germany; 4 University Center of Cardiovascular Science & Department of Cardiology, University Heart and Vascular Center Hamburg, University Medical Center Hamburg-Eppendorf, Hamburg, Germany; 5 School of Mathematics, Statistics, and Computer Science, University of KwaZulu-Natal, Pietermaritzburg, South Africa

## Abstract

Large-scale association analyses using whole-genome sequence data have become feasible, but understanding the functional impacts of these associations remains challenging. Although many tools are available to predict the functional impacts of genetic variants, it is unclear which tool should be used in practice. This work provides a practical guide to assist in selecting appropriate tools for variant annotation. We conducted a MEDLINE search up to November 10, 2023, and included tools that are applicable to a broad range of phenotypes, can be used locally, and have been recently updated. Tools were categorized based on the types of variants they accept and the functional impacts they predict. Sequence Ontology terms were used for standardization. We identified 118 databases and software packages, encompassing 36 variant types and 161 functional impacts. Combining only three tools, namely SnpEff, FAVOR, and SparkINFERNO, allows predicting 99 (61%) distinct functional impacts. Thirty-seven tools predict 89 functional impacts that are not supported by any other tool, while 75 tools predict pathogenicity and can be used within the ACMG/AMP guidelines in a clinical context. We launched a website allowing researchers to select tools based on desired variants and impacts. In summary, more than 100 tools are already available to predict approximately 160 functional impacts. About 60% of the functional impacts can be predicted by the combination of three tools. Unexpectedly, recent tools do not predict more impacts than older ones. Future research should allow predicting the functionality of so far unsupported variant types, such as gene fusions.

URL: https://cardio-care.shinyapps.io/VEP_Finder/.

Registration: OSF Registries on November 10, 2023, https://osf.io/s2gct.

**Supplementary Information:**

The online version contains supplementary material available at 10.1007/s00439-024-02670-5.

## Introduction

Whole-genome sequencing (WGS) has become precise and affordable on a large scale, and several cohorts now involve hundreds of thousands of subjects (Halldorsson et al. 2022; Taub et al. 2022; The All of Us Research Program Investigators 2019). Statistical analyses have associated diseases with common and rare variants (Povysil et al. 2019), and the GWAS Catalog currently contains more than half a million associations (Sollis et al. 2023). However, the causal mechanism behind most genetic associations is unclear and can take a long time to understand. For example, even for the best-replicated locus in cardiovascular disease, it took four years to unravel its function (Harismendy et al. 2011; Wellcome Trust Case Control Consortium 2007). To accelerate the understanding of biological function, a series of computational tools have been proposed in recent years.

In this work, we consider Variant Effect Predictors (VEPs) to be databases or software packages that predict the functional impacts of genetic variants. Each VEP is usually specialized in annotating one or a few categories of variants, such as single nucleotide variations (SNVs), indels, missense variants, or structural variants (SVs) (Geoffroy et al. 2018; Pagel et al. 2019; Rentzsch et al. 2019; Vaser et al. 2016). The variety of VEPs and their functionalities poses the challenge of choosing the appropriate tool for a specific task, a topic that has been addressed in non-systematic reviews (Katsonis et al. 2022; Tabarini et al. 2022). Some reviews summarize VEPs for one type of variant only (Abramowicz and Gos 2018; Glusman et al. 2017). Other articles focus on variation relevant to the American College of Medical Genetics and Genomics/Association of Molecular Pathology (ACMG/AMP) guidelines (Ghosh et al. 2017; Kassahn et al. 2014). All reviews have in common that their summary tables group functional information into a few categories, usually SNVs, indels, and SVs only. This categorization limits the search for VEPs suitable for other categories of variants, such as missense mutations or copy number variation.

This work aims to provide a systematic overview of the broad range of variant types and their functional impacts across VEPs. To this end, we systematically searched MEDLINE and investigated the possible input and output of each tool. The efficient selection of the most appropriate tool for a specific task can easily be accomplished using an interactive website.

## Methods

A systematic review was performed in accordance with the Preferred Reporting Items for Systematic Reviews and Meta-Analyses (PRISMA) guidelines (Page et al. 2021). The protocol was registered in OSF Registries on November 10, 2023 (10.17605/OSF.IO/S2GCT).

### Literature search

The literature search was conducted in the MEDLINE database. The search was restricted to articles published in English after January 1, 2014. This date was chosen to coincide with two milestones in genomics: the launch of the GRCh38 reference genome in December 2013 and the release of higher-throughput sequencing machines (Guo et al. 2017; Sheridan 2014). The search was performed on November 10, 2023, and results up to that date were included. The search query combined groups of terms related to variant, effect, prediction, and tools. Within each group, the terms were combined using the logical operator OR. The complete query is provided in Supplementary Table S1.

Articles containing the term “cancer” in the title were excluded to reduce the number of irrelevant hits and to find VEPs applicable across several diseases. We scanned the reference lists of review and benchmarking articles to retrieve additional eligible articles.

### Study selection

Included articles described a VEP, i.e., a tool accepting human genetic variants and predicting functional impacts. The list of exclusion criteria was made to ensure that tools were reliable, broadly applicable, accessible, scalable, and reproducible (Table 1). In cases where a tool appeared to be discontinued, generally indicated by a non-functional URL in the publication, we contacted the corresponding author for confirmation. Some authors supplied a working URL, which allowed us to reassess the publication against the other exclusion criteria. We removed tools not applicable to humans or without any documentation.

**Table 1:** Exclusion criteria

Not a database, tool, or score
Discontinued tool
Newer version available
Not applicable to humans
Not a VEP
No documentation
Preprint
Review or benchmarking publications
Not easily downloadable, e.g., web-only or GUI-only tool
Not completely free
Not supporting the GRCh38 genome build
Specific to a small number of genes
Specific to a disease
Not updated since January 1, 2020

Review and benchmarking articles were used to find additional eligible articles. However, only original work describing a VEP was included in this review. Web-only and GUI-only tools were deemed insufficiently reproducible and scalable and were thus excluded. In line with our accessibility requirement, tools requiring a fee were also excluded. Additionally, given the fast pace of progress in the field, we included only tools that support the GRCh38 genome build and were updated at least once since January 1, 2020. Tools that were specific to a small number of genes or a specific disease were excluded, as we were interested in the application of VEPs to a broad range of studies. If several versions of the tool existed, we only included the latest version, regardless of whether the latest version had an associated publication. Nevertheless, significant updates often coincided with a publication, such as dbNSFP v4 (Liu et al. 2020).

One author (CR) selected the studies based on the exclusion criteria (Table 1). First, titles were screened for eligibility. Second, articles were filtered based on the abstract. Third, the full text of the remaining articles was examined. Reasons for exclusion were recorded for each round.

### Data extraction

First, one author (CR) extracted the tool name, variant types, functional impacts, and operating system requirements from the included publications and their latest documentation. The URLs of tools with online capabilities were retrieved. Tools that required a high-performance computer were identified. Second, another author (LG) reviewed the extracted data to confirm the accuracy of the information from the publications and documentation. Divergences were resolved through discussion. For each article, the following characteristics were automatically retrieved: PubMed ID, title, authors, citation, first author, journal, year of publication, date of PubMed entry creation, PMCID, NIHMS ID, and digital object identifier.

Sequence Ontology terms were used to describe the variant types and functional impacts wherever applicable (Eilbeck et al. 2005). In case a Sequence Ontology term was unavailable to describe a particular variant type or functional impact, a new term was coined. For terms consistent with the structure of the Sequence Ontology, a request to create the new term was made on the Sequence Ontology GitHub page (https://github.com/The-Sequence-Ontology/SO-Ontologies/issues). Eighteen new terms were requested and are awaiting approval. Examples include “enhancer variant” and “promoter variant”. The full list of Sequence Ontology terms is provided in Supplementary Table S2.

### Data synthesis

Descriptive statistics were calculated for each tool, including the number of variant and functional impact categories. Linear regression was used to study the relationship between the number of functional impacts predicted by each tool and the date it was uploaded to the MEDLINE database.

### Software

All analyses used R version 4.2.2; all R scripts are attached as supplementary files and were uploaded to Zenodo (see section Code availability). A website was created with the shiny package (Chang et al. 2023).

## Results

**Fig. 1:**
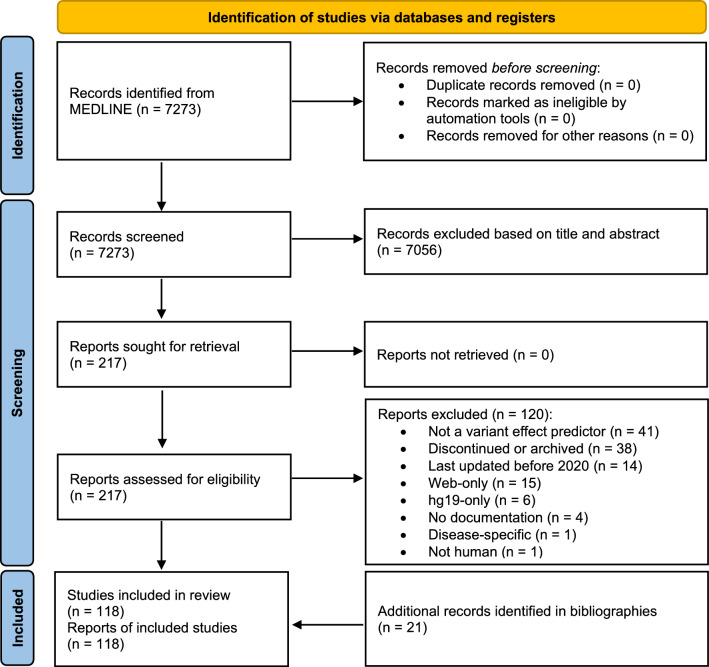
PRISMA flow diagram of the literature search and selection process

The MEDLINE query yielded 7273 records, of which 6514 were excluded after title screening (Fig. 1). Abstract screening excluded an additional 542 records, leaving 217 full-text articles for eligibility assessment. Detailed reading led to the exclusion of 120 articles. The most frequent reasons for exclusion were the fact that the work did not describe a VEP and the lack of maintenance. Examining references from benchmarking and review articles added 21 relevant publications. In total, this review encompasses 118 original articles, each covering a unique VEP, and references to all 118 articles are provided in Supplementary Table S3

### Overview of VEPs

**Fig. 2:**
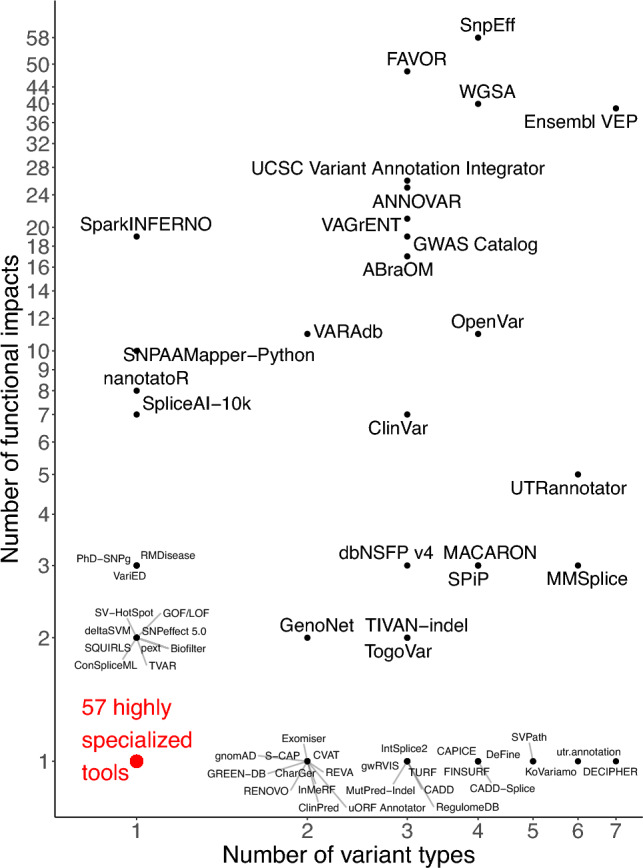
Scatter plot of the 118 variant effect predictors (VEPs). The number of functional impacts is plotted against the number of accepted variant types. To avoid overplotting, tools that annotate a single variant type and output only one functional impact type are collectively represented by a single red dot

The 118 VEPs differed in both the accepted variant types and the predicted functional impacts (Fig. 2). The number of accepted variant types per VEP ranged from one to seven. Seventy-three VEPs specialized in a single variant type, and two tools, Ensembl VEP and DECIPHER, accepted seven types (Bragin et al. 2014; McLaren et al. 2016). The number of predicted functional impacts per tool ranged from 1 to 58, and approximately two thirds (n = 82) predicted a single functional impact. SnpEff stood out as the VEP with the most predicted functional impacts (Cingolani et al. 2012). Some databases achieved many predicted impacts by aggregating predictions from multiple sources. For example, FAVOR and WGSA aggregated 48 and 40 annotations, respectively (Liu et al. 2016; Zhou et al. 2023).

The implementation of VEPs varied substantially. For example, AbraOM was a simple text file that lists variants and their functional impacts, while SparkINFERNO was a complex software package requiring a high-performance computing environment for execution (Kuksa et al. 2020; Naslavsky et al. 2017).

### Variant types

**Fig. 3:**
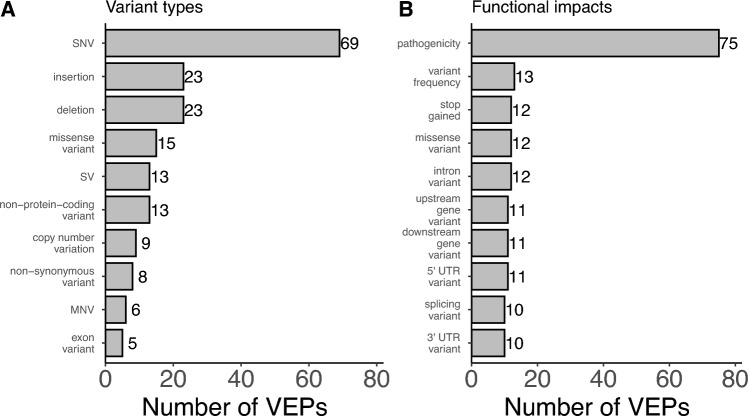
Barplots of the most commonly annotated variant types and predicted functional impacts. **A** Bars represent the number of tools accepting each of the 10 most commonly accepted variant types. **B** Bars represent the number of tools predicting each of the 10 most commonly predicted functional impacts

A total of 36 distinct variant types were accepted by VEPs, such as SNVs, indels, CNVs (Supplementary Table S4, Ding et al. 2023). Other acceptable inputs included variants falling in specific regions, such as splice sites, introns, exons, untranslated regions (UTRs), promoters, or enhancers. Despite the diverse range of variants being accepted across tools, some clinically and biologically important variants were missing. Gene fusions were unsupported by VEPs, both as input and predicted functional impacts. Furthermore, variant types were not equally represented across the tools (Fig. 3). While 69 VEPs accepted SNVs as input, 19 other variant types were accepted by only one VEP. For example, DECIPHER was the only database containing inversions and translocations (Bragin et al. 2014).

### Functional impacts

VEPs predicted 161 distinct functional impacts (Supplementary Table S5). Pathogenicity was the most common functional impact, predicted by 75 VEPs, because of its clinical relevance and use within ACMG/AMP guidelines (Fig. 3; Richards et al. 2015). Variant frequency, stop gain, and missense variant were the next most commonly predicted functional impacts (Fig. 3). Although variant frequency technically is not a functional impact, it has been reported by some databases and can provide insight into the evolutionary context and potential benignity of certain variants. Functional impacts are generally classified into several categories, and effects on the protein sequence, such as missense variants and frameshift indels, formed one well-studied class. Effects on splicing and regulatory elements, e.g., transcription factor-binding sites and enhancers, formed additional categories. Some tools predicted functional impacts not supported by other tools. For example, UTRannotator was the sole predictor of five specific changes in the open reading frames of 5′ UTRs (Zhang et al. 2021). Eighty-nine functional impacts were predicted by only one tool, making these tools indispensable for a study aiming to interpret those impacts.

Only Ensembl VEP, SnpEff, and VAGrENT used a controlled vocabulary, the Sequence Ontology, to describe functional impacts (Cingolani et al. 2012; McLaren et al. 2016; Menzies et al. 2015). However, none of the tools used the Sequence Ontology to describe variant types. Controlled vocabularies may also be used for phenotypes, such as the Experimental Factor Ontology in the GWAS Catalog or Human Phenotype Ontology for DECIPHER.

**Fig. 4:**
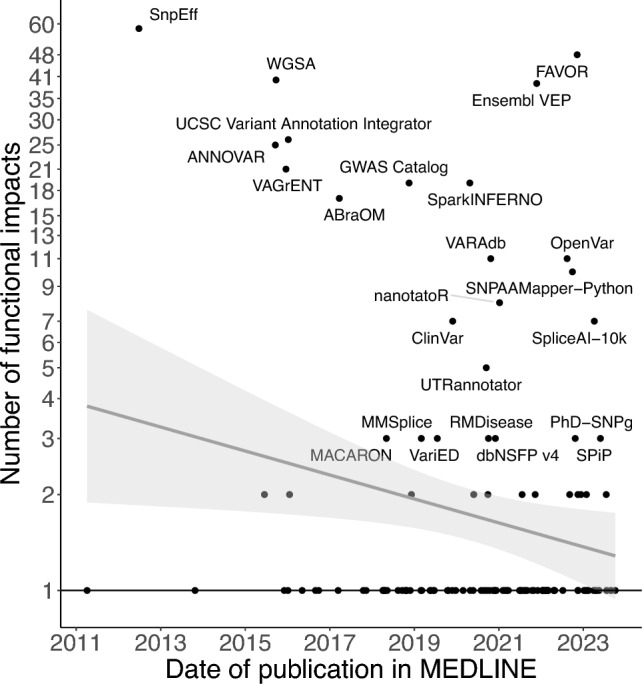
Number of predicted functional impacts annotated by each tool over time. Tools with  ≥ 3 predictions are labeled. The x-axis shows the day of publication in MEDLINE. The y-axis shows the number of predicted functional impacts in the most recent version of the tool. The grey line represents the least squares regression line. The light grey shaded area surrounding this line represents the 95% pointwise confidence intervals. The flat black line represents the quantile regression line at the median

Figure 4 displays the number of functional impacts by date of publication. While two aggregators (FAVOR and Ensembl VEP) had a publication in the last two years, SnpEff and WGSA were published more than seven years ago. The slope of the linear regression line was –1.03 (95% confidence interval –1.68, –0.37) functional impacts/year, which was significantly different from 0 (p = 0.002). As the assumption of linearity in this regression model is questionable, we also ran a quantile regression for the median. The quantile regression revealed a slope of 0 (95% confidence interval 0, 0) and an intercept of 1, indicating no change in the median number of predicted functional impacts over time. This result is due to the 82 VEPs that predict only one functional impact. The number of functional impacts presented in Fig. 4 does not necessarily correspond to the number predicted in that year. This discrepancy can occur because tools are updated, and we extracted data from the most recent documentation.

Supplementary Fig. S1 shows the number of variant types supported for the first time each year. There was no statistically significant upward or downward trend (p = 0.212).

### A Shiny app to find VEPs

To identify suitable VEPs, a Shiny app website can be used. The website features a searchable table listing the tools along with the specific variant types and functional impacts they address. Users can filter this table using Sequence Ontology terms relevant to their needs. They can also enter the number of VEPs they wish to implement. The site will then display the tools that maximize the number of impact predictions. For example, the top three tools, SnpEff, FAVOR, and SparkInferno, predict 99 different impacts, thus cover 61% of all possible impacts. In this combination, SnpEff covers 54 functional impacts covering changes in the coding sequence, UTRs, gene structure, regulatory regions, splicing, and others. FAVOR adds 36 annotations related to histone modifications, pathogenicity scores, and disease associations. SparkINFERNO adds 9 annotations related to non-coding RNAs and regulatory regions. Users may also filter VEPs according to the supported operating system and the availability of an online version of the tool. The website also includes a bibliography of review and benchmark articles. Benchmark studies that compare the performance of various tools provide valuable assistance in refining tool selection. One study's results, including sensitivity, specificity, positive predictive value, and negative predictive value, are accessible in a searchable table. REVEL is the best performer according to all four metrics (Ghosh et al. 2017). A recent benchmark study identified ClinPred and REVEL as the top pathogenicity predictors in this review, with the ranking of all 55 evaluated predictors accessible via the Shiny app (Livesey and Marsh 2023). While some tools performed better than ClinPred and REVEL, they were excluded from our review for not meeting the inclusion criteria. For example, ESM-1v was not peer-reviewed (Meier et al. 2021), and EVE and DeepSequence did not accept genetic variants (Frazer et al. 2021; Riesselman et al. 2018).

## Discussion

This systematic review identified 118 VEPs that together accepted 36 variant types and predicted 161 functional impacts. The functionalities of these numerous VEPs exhibited considerable diversity. Some VEPs accepted only one variant type, while others accepted up to 7. Similarly, some VEPs predicted a single functional impact, while others predicted up to 58. About half of these tools were highly specialized and predicted a single functional impact for one variant type. In contrast, SnpEff, FAVOR, and SparkINFERNO, could predict more than 40 functional impacts each. Using only these three VEPs covered 61% of the predictable functional impacts. Additionally, 75 tools predicted pathogenicity, making them usable as supporting diagnostic evidence according to the ACMG/AMP guidelines (Richards et al. 2015). To facilitate the selection of VEPs, we launched an interactive website that presents a list of tools according to user needs.

Out of 217 full-text articles analyzed, 38 VEPs have been completely discontinued, and another 14 have not been updated since 2019. The lack of maintenance of biological databases and tools is a recurring issue (Imker 2018), which causes difficulty for researchers who depend on these resources. Financial constraints and limited value may justify the discontinuation of a database. However, the database should be archived in a repository to ensure reproducibility (Imker 2018).

Our search was limited to MEDLINE and English language articles, potentially missing relevant studies in other databases or languages. To mitigate these limitations, we examined the references in review and benchmarking articles found by our search to find missed publications. Furthermore, we note that two recent reviews on rare non-coding variant annotation tools encompass 40 and 30 tools, respectively (Kuksa et al. 2022; Tabarini et al. 2022). One benchmarking paper covers 55 VEPs (Livesey and Marsh 2023). Thus, our review stands as the most comprehensive with 118 VEPs described.

In the screening process, we removed tools that were disease-specific, gene-specific, web-only, not updated since 2019, or rely on the hg19 genome build. While some tools may still be useful in specific contexts, we excluded them from our review to focus on broadly applicable, up-to-date, and scalable tools. While web-only databases are easily accessible, they lack the reproducibility, scalability, and privacy of downloadable VEPs.

Another limitation of our review is that it does not provide the total number of annotated variants for each tool. For example, while CADD scored each of the possible ∼ 9 billion human SNVs, the ABraOM database contained only 2.3 million variants. In fact, it is difficult to provide the precise number of annotated variants for each tool, as databases are subject to constant updates. Any number reported in the original publication is most likely outdated.

Adoption of a common standardized vocabulary would improve the comparison, integration, and discoverability of VEPs (Brookes and Robinson 2015). Only Ensembl VEP, SnpEff, and VAGrENT used a controlled vocabulary to describe functional impacts (Cingolani et al. 2012; McLaren et al. 2016; Menzies et al. 2015). In this review, we standardized the input and output terms used by each tool according to the Sequence Ontology (Eilbeck et al. 2005). This approach facilitates the search for tools in VEP Finder. For example, users interested in VEPs that accept 5′ UTR variants can choose ‘5_prime_UTR_variant’ from a drop-down menu, thereby avoiding confusion over non-standard terms. VEP Finder will then display all the tools that accept variants in 5' UTRs. The term ‘pathogenicity’ is widely used across the 75 pathogenicity predictors to describe variant impact, usually on a scale from ‘benign’ to ‘pathogenic.’ However, the inconsistent scale and vocabulary across tools, with some using terms like ‘neutral,’ ‘tolerated,’ or ‘deleterious,’ complicates direct comparisons. While most tools focus on disease relevance, some, such as SIFT, assess the effect on protein function (Vaser et al. 2016). The ACMG/AMP guidelines provide a standardized framework for defining pathogenicity concerning disease, but no similar classification guidelines exist for protein function. Thus, authors must clearly define what they mean by “pathogenicity” and how to interpret the scores.

Selecting suitable VEPs requires considering parameters beyond the accepted input and predicted output. Metrics, such as accuracy and precision, help in identifying tools with higher analytical performance (Livesey and Marsh 2023; Pejaver et al. 2022). Once the selection of VEPs has been made, guidance exists to interpret their outputs (Cheng et al. 2020). For diagnostic purposes, clinicians are advised to consult the ACMG/AMP guidelines to use VEPs (Richards et al. 2015), and for a limited number of genes, more detailed guidance on variant interpretation is available (Fortuno et al. 2021; Lee et al. 2018).

### Future research

This review revealed that no VEP accepts gene fusions as input. This gap may be due to their lower frequency in the human population and because of the limitations of second-generation sequencing technologies. However, their clinical importance calls for support soon (Nelson et al. 2017). New variant types were regularly supported (Fig. S1). Should this trend continue, more variant types will likely receive support in the coming years.

The absence of a benchmarking study assessing all 75 pathogenicity predictors highlights the difficulty of this endeavor. A meta-analysis of existing studies could shed light on the best-performing VEPs and might discriminate between the many pathogenicity predictors. This analysis would need to account for the variability in the sets of tools and testing datasets used across different studies.

To maximize the utility of VEPs for clinical and research purposes, further advancements are required to extend predictions specific to isoforms, tissues, and traits to more variants. Such functionality will enhance our understanding of variant effects and facilitate their experimental validation. Moreover, developing trait-specific pathogenicity scores is essential because certain variants may be pathogenic for one disease but benign or even advantageous for another (Taylor et al. 2012). Furthermore, to facilitate interoperability between different tools, we also advocate the use of controlled vocabularies to describe phenotypes (Kohler et al. 2021; Malone et al. 2010). We aim to perform a bigger update of the VEP Finder once per year and to do regular update after user input and evidence.

VEPs predicting many functional impacts, such as SnpEff, FAVOR and WGSA, represent a potential solution to the problem of tool choice. Nevertheless, the rapid evolution of the field necessitates continuous updates to keep them up to date. Furthermore, we expect specialized tools to be continuously released (Fig. 4). Consequently, systematic reviews on VEPs will be needed regularly.

## Conclusion

A staggering 118 tools were available to predict approximately 160 functional impacts that ranged from molecular to phenotypic effects. About 60% of these impacts could be predicted by combining just three tools. Unexpectedly, recent tools did not necessarily predict more impacts than older ones. Despite the vast diversity of VEPs, some genetic variants were not yet supported and should be the object of future research.

The abundance of available options can complicate the tool selection process. However, this challenge is mitigated by the Shiny app developed in this review. The app enables users to filter tools based on their specific needs, narrowing down the list of suitable options.

### Supplementary Information

Below is the link to the electronic supplementary material.

Supplementary file1: Fig. S1. Bar plot of the number of variant types that receive support by a VEP for the first time each year (p linear regression = 0.212) (PDF 5 KB)

Supplementary file2: Table S1. Query performed in PubMed on November 10, 2023 (XLSX 12 KB)

Supplementary file3: Table S2. Terms used in this review to describe variant types and functional impacts, including Sequence Ontology terms (XLSX 14 KB)

Supplementary file4: Table S3. List of publications included in this systematic review. The table lists the PubMed ID, tool name, list of variant types, list of functional impacts, a note, publication title, authors, citation, journal, year of publication, creation date of the PubMed entry, PubMed Central ID, NIHMS ID, and DOI. SO terms related to variation types are listed alphabetically and separated by semicolons under the column “Variant types”. Similarly, SO terms related to predicted functional impacts are listed under the column “Functional impacts” (XLSX 39 KB)

Supplementary file5: Table S4. List of variant types and number of VEPs supporting each type (XLSX 10 KB)

Supplementary file6: Table S5. List of functional impacts and number of VEPs predicting each impact (XLSX 13 KB)

Supplementary file7 (ZIP 11 KB)

## Data Availability

The VEP Finder website is freely available at https://cardio-care.shinyapps.io/VEP_Finder/.

## Code availability

The scripts for data wrangling, generating plots, and running linear models are stored on Zenodo and can be accessed at 10.5281/zenodo.10255446. They are also available in the Supplementary zip file. The code for the Shiny app is accessible at https://github.com/CristianRiccio/VEP_Finder.
