# Supplementary figures and images for: Variant effect predictors: a systematic review and practical guide
Source: Hum Genet. 2024 Apr 4;143(5):625–34. doi: 10.1007/s00439-024-02670-5 (PMC11098935; doi:10.1007/s00439-024-02670-5)

Number of variant types supported for the first time

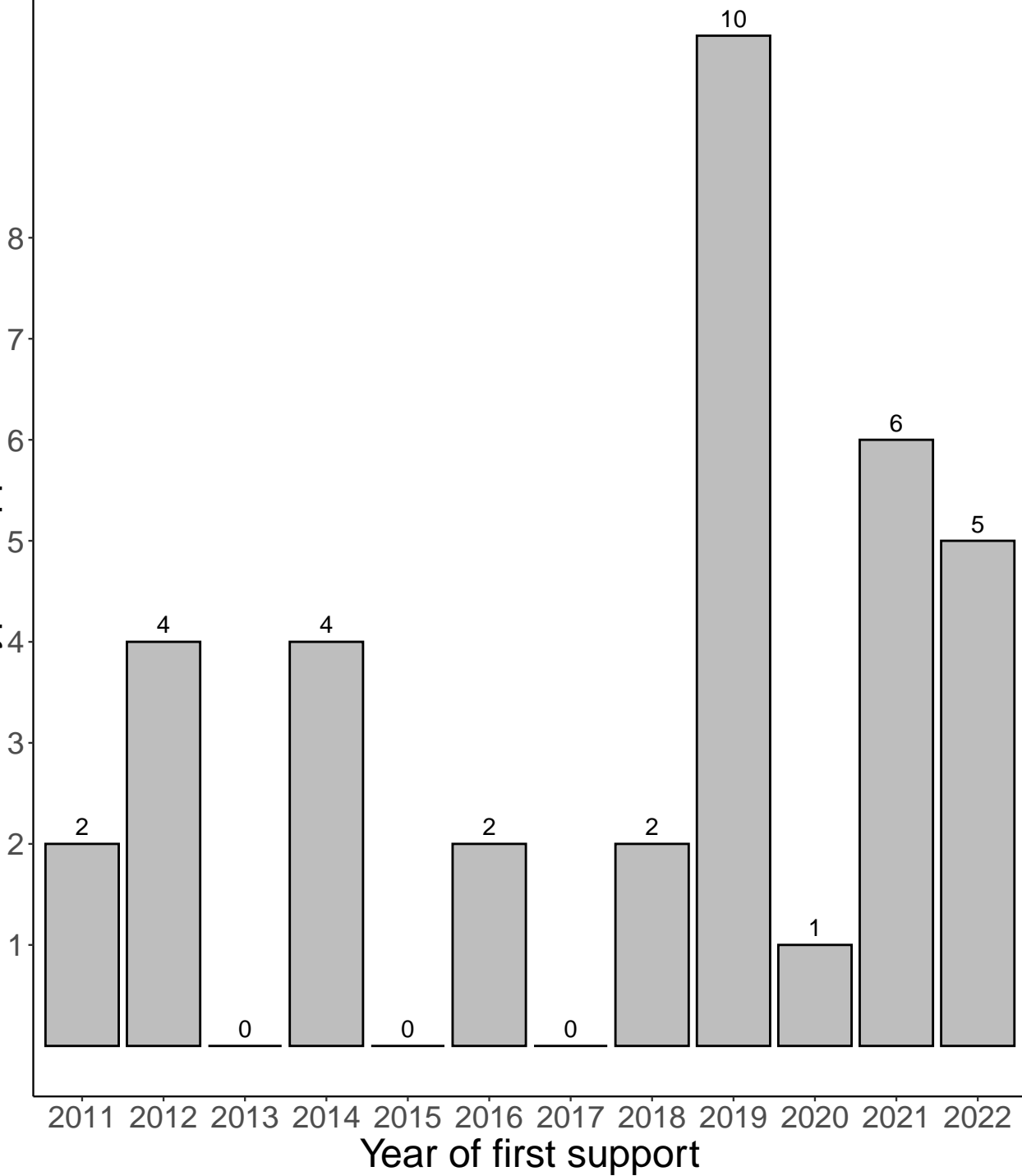

Supplement: Supplementary file 1 — Supplementary file1: Fig. S1. Bar plot of the number of variant types that receive support by a VEP for the first time each year (p linear regression = 0.212) (PDF 5 KB) [file 439_2024_2670_MOESM1_ESM.pdf]
